# A microfluidic electrochemical immunosensor for detection of CEA and Ki67 in 3D tumor spheroids

**DOI:** 10.1016/j.mtbio.2025.101768

**Published:** 2025-04-12

**Authors:** Sujin Kim, Seonyeop Kim, Chanjin Ko, Wonseok Lee, Hwan Drew Kim

**Affiliations:** a Department of Polymer Science and Engineering, Korea National University of Transportation, 50 Daehak-ro, Chungju, 27469, Republic of Korea; b Department of IT-Energy Convergence (BK21 Four), Chemical Industry Institute, Korea National University of Transportation, 50 Daehak-ro, Chungju, 27469, Republic of Korea; c Department of Electrical Engineering, Korea National University of Transportation, 50 Daehak-ro, Chungju, 27469, Republic of Korea; d Department of Biomedical Engineering, Korea National University of Transportation, 50 Daehak-ro, Chungju, 27469, Republic of Korea

**Keywords:** Electrochemical sensor, Immunosensor, Microfluidics, Cancer biomarker, Carcinoembryonic antigen

## Abstract

Microfluidic chip-based electrochemical sensors have been developed to detect cancer biomarkers and monitor changes in the tumor microenvironment. However, the limitation of detecting only a single biomarker restricts their utility as accurate diagnostic tools. Simultaneous detection of multiple tumor biomarkers is important for early diagnosis of cancer. In this work, we report the development of a microfluidic-based electrochemical immunosensor platform capable of simultaneously observing multiple biomarkers expressed by three dimensions (3D) cell spheroids. The sensor platform employs a nanocomposite electrode material consisting of gold nanoparticles and carbon nanotubes, which enables sensitive and selective detection. The sensor was fabricated using 3D and printed circuit boards (PCB) printing techniques, demonstrating the feasibility of cost-effective manufacturing. The developed platform was able to quantitatively detect two key cancer biomarkers, carcinoembryonic antigen (CEA) and Ki67, with limits of detection of 0.97 ng/mL for each. Furthermore, the sensor was successfully utilized to observe the knockdown of these biomarkers, showcasing its potential for both diagnostic and therapeutic monitoring applications. These results suggest that the presented electrochemical sensor platform provides a promising lab-on-a-chip technology for comprehensive 3D cell spheroid-based cancer biomarker analysis, which could have significant implications for future clinical diagnostics and personalized medicine.

## Highlights

• Microfluidic electrochemical immunosensor for rapid, sensitive CEA/Ki67 detection in 3D tumor spheroids.
• Low detection limit for precise biomarker quantification achieved.
• Validated on murine/human CRC lines, detecting biomarker changes post-gene knockdown.
• Potential for multiplexed detection, monitoring therapy response in cancer research shown.

## Graphical abstract

**Figure:**
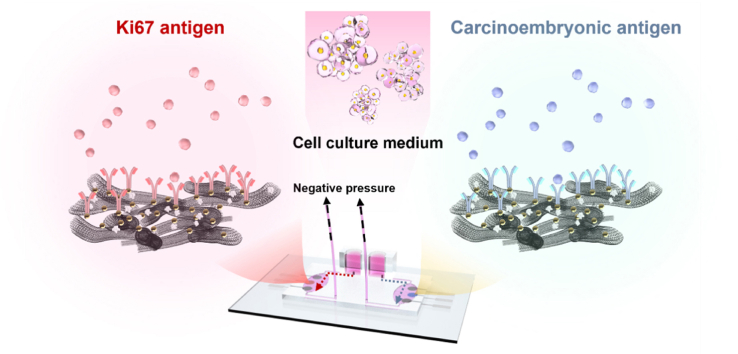

## 1 Introduction

Cancer remains a leading cause of death worldwide, posing a significant obstacle to increasing human life expectancy [1,2]. This burden is exacerbated by an aging global population and the rising prevalence of modifiable risk factors such as smoking, unhealthy diets, and physical inactivity [1,3,4]. The types of cancer come in various forms depending on living organs such as prostate cancer, breast cancer, colon cancer, stomach cancer and skin cancer. The incidence of cancer is predicted by global demographics to generate about 420 million new cancer cases every year by 2025 [5]. Colorectal cancer (CRC), the third most common cancer diagnosis in the United States, exemplifies this trend, with increasing incidence observed even in younger populations [6]. Despite advances in treatment modalities, including surgical resection, radiotherapy, and targeted therapies, CRC mortality remains high [3,4]. CRC is a long-term process that spans decades, and the stages of CRC can be summarized into a total of five. Stage 0, only found on the innermost inner wall of the large intestine called the mucous membrane; stage 1, where the tumor spreads beyond the inner membrane; and stage 2, where it extends through the thick outer muscle layer, does not spread to lymph nodes that act as filters as small organs of parts of the immune system. However, it spreads from the outside of the large intestine to the lymph nodes, and in stages 3–4, it spreads to the body, such as the liver and lungs. CRC, in which symptoms appear only in the progressive stage, shows a difference in treatment effectiveness and survival rate depending on the stage of diagnosis. This underscores the critical need for early detection and intervention to improve patient prognosis and quality of life [7,8].

Traditional two-dimensional (2D) cell cultures cultured at the bottom of cell culture plates have limitations in summarizing complex tumor microenvironments, which impede accurate assessment of tumor behavior and drug response. Three-dimensional (3D) cell spheroid cultured without attachment to the bottom provides a promising alternative that provides biomimetic models that better reflect *in vivo* conditions [9,10]. These 3D models enable the investigation of critical aspects of cancer progression, including cell-cell interactions, growth kinetics, invasion, and therapeutic response [11,12]. Furthermore, 3D spheroids are amenable to advanced analytical techniques, such as imaging, genetic analysis (e.g., Polymerase chain reaction; PCR), and protein expression profiling (e.g., Enzyme-linked immunosorbent assay; ELISA-based immunosensors) [13,14]. While ELISA offers sensitive and quantitative protein detection, its inherent complexity and time-consuming nature limit its high-throughput applications [12]. Electrochemical sensors have emerged as a powerful alternative, offering rapid and sensitive detection of biomarker expression in 3D spheroids [13,15]. Previous studies have demonstrated the feasibility of electrochemical detection for biomarkers such as HER2 and PD-L1 [16,17]. And electrochemical sensor can be integrated with microfluidic devices for the multiplex analysis of biomarkers [18]. Microfluidic-based electrochemical sensors have benefits of high sensitivity, short detection time and low reagent consumption [19].

**Scheme 1:**
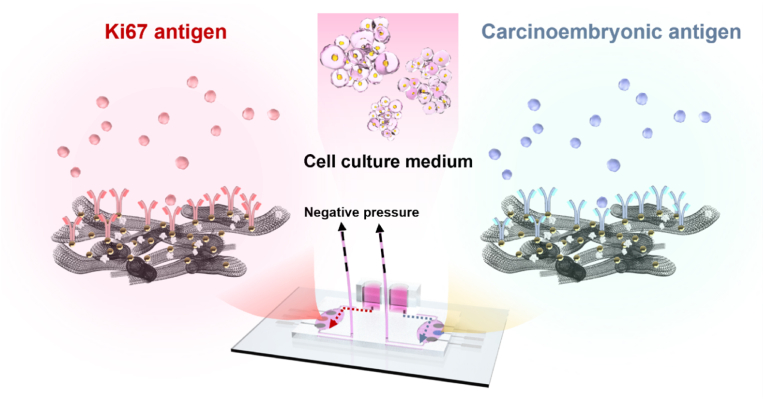
Schematic illustration: Detection process of antigens from cell culture medium with anti-Ki67 and anti-CEA antibody functionalized immunosensor.

Thus, we developed a novel microfluidic-based electrochemical immunosensor platform capable of simultaneous detection of multiple CRC-related biomarkers expressed by 3D cell spheroids. This platform integrates 3D-printed microfluidic layers with nanocomposite electrodes fabricated using a PCB printer, enabling high-throughput analysis of clinically relevant biomarkers such as Ki67 and CEACAM5. We validated the platform's performance using 3D spheroids generated from various CRC cell lines (CT-26, HT-29) and mesenchymal stem cells (MSCs), confirming its ability to detect changes in biomarker expression following gene knockdown with siRNA. This pilot study demonstrates the potential of our microfluidic-based electrochemical immunosensor platform as a valuable tool for cancer research and diagnostics (Scheme 1).

## 2 Experimental section

### 2.1 Materials and reagents

Multi-walled carbon nanotube solution (MWCNTs, 95 %, OD: 5–15 nm, 3 wt% in water) (US7907, US) was purchased from Nano solution Inc. Tetrachloroauric(III) acid (520918, US), phosphate buffered saline (P4417, US), Potassium hexacyanoferrate(III) (K_3_Fe(CN)_6_) (244023, US), Potassium hexacyanoferrate(II) trihydrate (K_4_Fe(CN)_6_·3H_2_O) (455989, US), potassium chloride (KCl) (P3911, US), sodium citrate tribasic dihydrate (S4641, US) and 2-propanol (190764, US) were purchased from Sigma Aldrich. Polydimethylsiloxane (Sylgard 184, US) and curing agents were purchased from Dow corning. E-shell 300 resin ink (Germany) was purchased from EnvisionTEC (Germany). Carcinoembryonic antigen (CEA) (CEACAM5-3200H, US), Ki67 antigen (MKI67-245H, US) and CA19-9 antigen (CA19-9-129H, US) were purchased from CREATIVE BIOMART. CEA antibody (ab133633, abcam, UK) and Ki67 antibody (14-5699-82, Invitrogen, US) were purchased from OriGene Technologies Inc (US). The conductive ink (Conductor-III, Voltera) (1000681, Canada) was applied for electrode printing. The screen-printed carbon electrode was purchased from Metrohm AG (DRP-C110, Switzerland). The deionized water (DI water, 18.2 MΩ•cm) was used to fabricate all solutions. Glass substrate (S9213, Japan) was purchased from MATSUNAMI. Phosphate-buffered solutions (PBS, pH 7.2) were prepared by diluting phosphate-buffered saline with 200 mL of DI water.

### 2.2 Apparatus

All electrochemical measurements were carried out by using CS2350 Bipotentiostat (Ivium technologies) and compactstat (Ivium technology, Netherland). A vacuum plasma system (COVANCE, femto science) was used for oxygen (O_2_) plasma treatment on a glass substrate to attach the PDMS microfluidic layer. A Voltera V-ONE printer was used to print electrodes. SLA 3D printer (Micro plus HD, EnvisionTEC) was used to fabricate the resin master mold for the microfluidic layer.

### 2.3 Preparation of AuNP solution

The AuNP solution was synthesized with common method [14,20]. The 100 mL of gold chloride solution (1 mL of 1 wt% HAuCl_4_ solution and 99 mL of DI water) was refluxed at 100 °C to boiling under magnetic stirring with 200 rpm. 2.5 mL of 1 wt% sodium citrate solution was added rapidly to gold chloride solution. Subsequently, the color of the solution turned wine red, indicating the formation of the Au nanoparticle (AuNP) solution. After that, 1 mL of AuNP solution was centrifuged at 10,000 rpm for 15 min. Then, 900 μL supernatant was removed from the solution to enrich the concentration of AuNP solution [21]. As a result, 100 μL of highly concentrated, deep wine-red AuNP solution was obtained, as shown in Fig. S1.

### 2.4 Fabrication of immunosensor

The electrode was designed using AutoCAD and converted to Gerber file via Fusion 360 program. The designed electrode was printed using Voltera V-ONE PCB printer onto a glass substrate (76 × 52 mm) using conductive ink (Conductor-III ink). The conductor III ink is an Ag-based conductive ink for PCB printing characterized by high conductivity and rapid curing, making it suitable for electrode printing. The printing nozzle had an inner diameter (ID) of 230 μm and an outer diameter (OD) of 420 μm. The circuit resolution was 400 μm. The nozzle travel distance was set to 1.5 mm, indicating that the nozzle slightly rose and descended after every 1.5 mm of ink printing to reapply pressure and ensure uniform electrode printing. The printed ink was cured by the implemented heater of the printer with a curing temperature of 140 °C. The MWCNTs solution was drop-casted on the end of the printed circuit with the amount of 2 μL to fabricate the 3-electrode system (including working, reference and counter electrode). All electrodes were composed with the same material (MWCNTs) to miniaturize the fabrication process of the electrode and to prevent polarization [22], which results in potential drops and reduced sensitivity at the electrode-electrolyte interface. Then, drop-casted MWCNT droplets were dried at 70 °C for 15 min. The diameter of dried MWCNTs droplets was approximately 4 mm. Furthermore, 3 μL of AuNP solution was drop-casted onto the working electrode and dried at 70 °C for 15 min. After cooling down the electrode for 20 min, 50 μg/mL of anti-CEA antibody anti-Ki67 antibody were drop-casted onto the working electrode with the amount of 3 μL. The antibody-deposited electrode was kept under 4 °C overnight. Then, the electrode was washed gently with PBS solution and blocked by drop-casting BSA solution (1 wt%) with 3 μL for an hour. After blocking, the electrode was washed in PBS.

### 2.5 Composition of microfluidic layer and chamber

The microfluidic layer was designed with the reservoir in the middle of the layer. The master molds of the microfluidic layer and chamber were printed by the SLA 3D printer with resin ink. The SLA 3D printing techniques are widely used for the fabrication of miniaturized analytical devices and molds, including microfluidic channels and medical equipment. However, a major limitation of this method is that commercially available resin inks for SLA 3D printing inhibit PDMS curing when the resin structure is used as a mold. For this reason, we selected E-shell 300 resin ink for the master mold of microfluidic layer, as it offers high biocompatibility and processability [23]. The master mold for the microfluidic layer was designed as a 3D modeling file (.stl) and then printed with a resolution of 60 μm in the XY-axis and 50–150 μm in Z-axis. The UV exposure time was set to 18,000 ms and 11,000 ms for the initial burning and main exposure process, respectively (Fig. S2A). The height and total volume of microfluidic layer and chamber were 500 μm/56.09 mm^3^and 10 mm/702.74 mm^3^, respectively (Fig. S2B). The printed master molds were washed with 2-propanol to remove the remaining uncured resin ink. The washed master molds were treated with a UV lamp for 1 h and then dried at 120 °C for 1 h to detach the PDMS layer from the master molds without collapsing. The surface-treated master molds were fixed onto the surface of a petri dish, and a mixture of PDMS pre-polymer and curing agent (10:1, w/w) was poured into the molds. After degassing for an hour, the pre-polymer was cured in the oven at 70 °C for 3 h. The cured PDMS microfluidic layer was removed from the master mold and punched with a biopsy punch (1 mm diameter) to configure the inlet and outlet on the fluidics layer. A cured PDMS chamber with two ellipse-shaped holes (1.3 × 0.6 × 1 cm^3^) for containing the solution was cut into 1.8 × 1.8 × 0.8 cm^3^ size. The microfluidic layer and chamber were attached to the immunosensor with O_2_ plasma treatment at 150 W for 60 s. As a result, microfluidic-based electrochemical sensing platform with two electrode system was fabricated, as shown in Fig. S2C.

### 2.6 Electrochemical measurement

The electrochemical properties of the immunosensor were analyzed using cyclic voltammetry (CV) and linear sweep voltammetry (LSV) methods with the current response. The electrochemical measurements were conducted using an electrolyte solution containing 0.01 M PBS (pH 7.2) containing 5 mM of [Fe(CN)_6_^3−^/^4-^] with 0.1 M KCl at room temperature condition. The immunosensors were incubated in the CEA and Ki67 antigen solution with various concentrations for 45 min at 4 °C by injecting solutions into a microfluidic layer with volume of 56.1 mm^3^. The potential voltage range was set from −1.5 V to 1.0 V for electrochemical property analysis and from −0.4 to 0.6 V for electrochemical optimization process in CV measurements. The potential voltage range was set from −0.4 to 0.6 V for CV and −0.5 to 0.5 V for LSV measurement at a scan rate of 50 mVs^−1^. All electrochemical measurements were conducted at room temperature condition. The immunosensors were stored at 4 °C when not in use for measurements.

### 2.7 Optimization of immunosensor

To fabricate a highly sensitive immunosensor, electrode optimization was carried out for both MWCNTs and AuNP. Firstly, electrochemical properties of various concentrations of MWCNTs with 0 (bare), 2, 2.5, 3 and 3.5 wt% were evaluated using CV method (ranging from −0.4 V to 0.6 V) onto SPCE (screen printed carbon electrode). For each condition, 2 μL of MWCNTs solution was drop-casted onto the working electrode of SPCE, and three separate electrodes were prepared per concentration for optimization process. The oxidation peak currents were normalized to the average value of bare electrodes. The normalized oxidation peak current values were 2.63 ± 0.42, 3.14 ± 0.28, 3.48 ± 0.30 and 3.58 ± 0.23 for 2, 2.5, 3 and 3.5 wt% of MWCNTs applied SPCE, respectively (Fig. 3A). The oxidation peak current was dramatically increased by 163 % from 0 to 2 wt% of MWCNTs concentration, however, the variation gradually decreased as the MWCNTs concentration increased and plateaued at 3 wt%. For AuNP, we compared bare electrode (no AuNP), 1-fold (pristine), 5-fold, 10-fold and 15-fold concentrated AuNP solution using SPCE under the same measurement conditions. The normalized oxidation peak current values were 1.07 ± 0.02, 1.19 ± 0.01, 1.27 ± 0.02 and 1.29 ± 0.02 for the 1-, 5-, 10- and 15-fold concentrated AuNP solution, respectively (Fig. S3). The peak current gradually increased with increasing concentration and saturated at 10-fold concentrated AuNP with minimal variation (2.01 %). Accordingly, 3 wt% MWCNTs and 10-fold concentrated AuNP solution were selected as the optimal conditions. Furthermore, antibody concentration optimization was performed for both anti-CEA and anti-Ki67 antibody using fabricated immunosensors. The various concentrations of antibodies (0, 25, 50 and 100 μg/mL) were immobilized onto the working electrode with the amount of 3 μL and stored overnight at 4 °C and gently washed with PBS solution to remove unbound antibodies. The LSV method was used with −0.5 V–0.5 V potential range to evaluate the electrochemical property change induced by antibody immobilization. The average peak currents of 0 μg/mL of antibody applied AuNP@MWCNT electrode (bare) were set as the control value for normalization. In the case of anti-CEA, the normalized peak currents were 0.93 ± 0.04, 0.88 ± 0.04 and 0.87 ± 0.05 for 25, 50 and 100 μg/mL, respectively (Fig. S4A). The peak current value decreased as the concentration of antibody increased. This result was due to the hindered electrode surface by antibodies, which are non-conductive bio molecules and have no electron transfer properties. Furthermore, this phenomenon can contribute to larger amount of antigen capturing [24]. The peak current was decreased by −6.88 % from 0 to 25 μg/mL, −5.69 % from 25 to 50 μg/mL and −1.24 % from 50 to 100 μg/mL. The variation of peak current was saturated from 50 μg/mL, thus, the optimum concentration of anti-CEA antibody was set to 50 μg/mL. Additionally, In the case of anti-Ki67, the normalized peak currents were 0.91 ± 0.03, 0.86 ± 0.06 and 0.85 ± 0.05 for 25, 50 and 100 μg/mL, respectively (Fig. S4B). The peak current was decreased with −8.82 % from 0 to 25 μg/mL, −5.62 % from 25 to 50 μg/mL and −1.17 % from 50 to 100 μg/mL. Similar to anti-CEA, the variation of peak current against various anti-Ki67 concentrations was saturated from 50 μg/mL. From this result, the optimum concentration of anti-Ki67 antibody was set to 50 μg/mL, the same as that of the anti-CEA antibody.

### 2.8 Cell culture

CT-26 was purchased from Korean Cell Line Bank (74364). HT-29 was purchased from ATCC (HTB-38). And mesenchymal stem cells were obtained from Seoul National University. CT-26 and HT-29 were cultured in Dulbecco's modified Eagles medium (DMEM) (#1000136026, STEMCELLTM, Canada) supplemented with 10 % fetal bovine serum (26140079, Gibco, US) and 1 % penicillin-streptomycin-glutamine (10378016, Gibco, US). Mesenchymal stem cells (MSC) were cultured in MesenCult™ Proliferation Kit (Human) (ST05411, STEMCELL™, Canada) supplemented with 10 % fetal bovine serum and 1 % penicillin-streptomycin-glutamine. All cells were cultured in a 5 % CO_2_/95 % O_2_ incubator at 37 °C, supplemented with fresh medium every 2–3 days.

### 2.9 Fabrication of spheroids

To simulate 3D-type tumors, tumor spheroids were manufactured using a platform called LabSphero™ (2035080, LabToLab, Korea). Prior to seeding the cells, the LabSphero™ was washed three times with ethanol (000E0219, SAMCHUN, Korea) and once with phosphate-buffered saline (PBS) (ST37350, STEMCELL™, Canada). CT-26 cells, HT-29 cells and MSCs were seeded at 1 × 10^6^ cells/dish cell density. Cells were cultured within the platform for 2 days, formed spheroids, and obtained on the last day. The number of single cells in the grid and the diameter and aspect ratio of the spheroid were quantified using ImageJ software (NIH, USA).

### 2.10 Immunofluorescence staining of spheroids

CT-26 cells, HT-29 cells, and MSCs were made into spheroid form by LabSphero™. Spheroids of each of the three cell types were obtained on day 2 of LabSphero™ culture and attached to gelatin-coated coverslips for microscopic observation. The spheroid was fixed using 3.6 % paraformaldehyde (PFA) (47608, Sigma-Aldrich, Switzerland) for 10 min and then washed with PBS. To increase the permeability, it was incubated with 0.5 % triton-X (T8787, Sigma-Aldrich, USA) solution for 15 min and then washed. Then, it was incubated at room temperature for 1 h in a solution where 0.2 % bovine serum albumin (BSA) (A7906, Sigma-Aldrich, US) and 0.1 % triton-X solution were mixed in a 1:1 ratio. After being washed, the primary antibody was incubated overnight at 4 °C in a solution mixed in PBS at a ratio of 1:500. After washing three times with 0.1 % triton-X solution, it was cultured for 1 h in a dark at room temperature using a secondary antibody. After washing three times, it was incubated for 5 min with 4′,6-diamidino-2-phenylindole (DAPI) (D1306, Invitrogen, US) in the dark at room temperature, and then washed and observed for spheroid through a fluorescence microscope. The fluorescence expression regions of the spheroids were quantified using ImageJ software. Ki-67 (14-5699-82) was purchased from Invitrogen. (ab133633) was purchased from Abcam. Also, the secondary antibodies Alexa-Fluor 594 (ab150080) and Alexa-Fluor 488 (ab150113) were purchased from Abcam.

### 2.11 Flow cytometer

CT-26 cells, HT-29 cells, and MSCs were immunofluorescent staining in a suspended condition. Washing was carried out after 5 min down at 1500 rpm at 4 °C using a centrifuge. Subsequently, it was dispersed in 100 μL PBS. Expression measurements were measured with a NovoCyte flow cytometer (NovoCyte 1000, Agilent Technologies, China) and analyzed with the NovoExpress program.

### 2.12 ELISA

A spheroid-cultured medium was collected to analyze the proteins within the culture medium. To remove cells and debris within the medium, the culture medium was filtered through a 200 μm syringe filter. For analysis, the Human Ki67 ELISA Kit (ab253221, Abcam, UK) and Human CEA ELISA Kit (244012424, Thermo Fisher, USA) were used. A 450 nm wavelength band was used to measure absorbance through a microplate reader (Infinite 200 Pro, Tecan, Swiss). Protein concentrations in each sample were calculated according to standard curves.

### 2.13 RT-PCR

RNA was extracted through PURY RNA Plus (P2030-050, GenDEPOT, USA), and RNA concentration was measured with a microplate reader. In addition, cDNA was synthesized by PrimeScript™ RT Master Mix (RR036A, TaKaRa, China), and synthesis was performed on VeritiPro™ Thermal Cycler, 96-well (A48141, Thermo Fisher, Korea). RT-PCR was performed on a QuantStudio 3 Real-Time PCR Instrument (A28132, Thermo Fisher, Singapore) using a Power SYBR® Green Real-Time PCR Master Mix (4367659, Thermo Fisher, UK). The genes measured gene amplification of *GAPDH*, *Mki67*, and *CEACAM5*, and the results were calculated as 2^ (-ΔΔ Ct) for *GAPDH*. The primer sequences used in the study are shown in Table S1.

### 2.14 Statistical analysis

Quantitative results were displayed in the form of mean ± standard deviation. One-way ANOVA and two-way ANOVA of Šídák's multiple comparisons test were used to identify significant differences between experimental groups. *∗p* < 0.05, *∗∗p* < 0.01, *∗∗∗p* < 0.001, *∗∗∗∗p* < 0.0001.

## 3 Results and discussion

### 3.1 Characterization of the electrochemical immunosensor at the microfluidic platform and *in vitro* detection of Ki67 and CEA

**Fig. 1:**
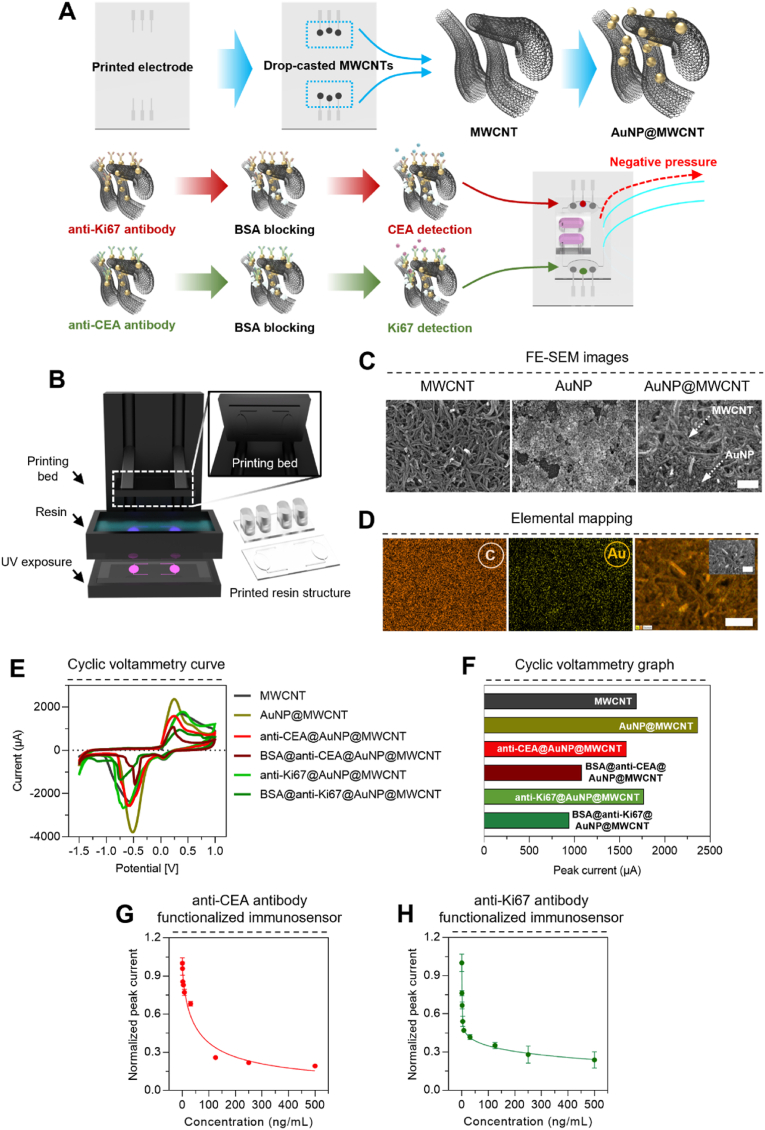
Characterization of the electrochemical immunosensor at the microfluidic platform and *in vitro* detection of Ki67 and CEA. Schematic illustration: (A) fabrication process of anti-CEA and anti-Ki67 antibody functionalized immunosensor. (B) Printing process of resin master molds for microfluidic layer and chamber fabrication. (C) FE-SEM image of MWCNT, AuNP and AuNP@MWCNT. (D) Elemental mapping of MWCNT, AuNP and AuNP@MWCNT. (E) Cyclic voltammetry graph of immunosensor in each fabrication step (F) Peak current of each fabrication step. Normalized peak current of (G) anti-CEA antibody and (H) anti-Ki67 antibody functionalized immunosensor after incubating in various concentrations of antigen for 35 min (n = 3). Scale bar: (C) 200 nm, (D) 250 nm.

To enable quantitative detection of the cancer biomarkers CEA and Ki67, we developed a microfluidic-based electrochemical immunosensor platform (Fig. 1A–B) using MWCNT and AuNPs. The CNTs (carbon nanotubes) have a high mechanical strength, better electrical conductivity and strong film-forming potential, which are suitable for constructing the electrode for the electrochemical sensor. Additionally, its biocompatible and bioactive properties have advantages for immobilizing the biomolecules to detect specific biomarkers [25]. Furthermore, its ability to conjugate with metal or organic components allows it to from AuNP@MWCNTs nanocomposites for sensitive detection of CEA and Ki67 antigen [26]. The AuNP was selected for constructing working electrode due to its high chemical stability, large surface to volume ratio, high biocompatibility and exceptional electrical conductivity which enables amplifying the electric signal and facilitates integration with antibodies and MWCNTs in electrochemical measurements [27]. The morphological characterization of the Au nanoparticle@multi-walled carbon nanotube (AuNP@MWCNT) working electrode via FE-SEM revealed a well-defined network structure, with MWCNTs providing a high surface area scaffold and AuNPs uniformly distributed throughout (Fig. 1C). Analysis of the AuNPs indicated an average size of 21.6 ± 3.3 nm. EDS mapping further confirmed the homogeneous distribution of AuNPs within the MWCNT matrix (Figu 1D and S5). This intercalation of AuNPs within the MWCNT network enhances electrode conductivity, leading to improved electrochemical signal transduction. Moreover, the AuNPs facilitate efficient antibody immobilization due to its large surface area and high biocompatibility. Additionally, AuNPs could increase conductivity and electron transfer property of the electrode, further contributing to the sensor's performance [28]. Meanwhile, the fabrication process involved a critical step to preserve antibody functionality during device assembly. Specifically, oxygen plasma treatment, essential for bonding the polydimethlsiloxane (PDMS) microfluidic layer to the electrode substrate, can potentially damage the immobilized antibodies on the working electrode. To mitigate this, we employed a shadow mask (SUS-430 stainless steel) to shield the antibody-functionalized regions during plasma treatment (Fig. S6A) [29]. Control experiments conducted without the shadow mask confirmed its effectiveness in preserving antibody activity, as evidenced by significantly higher peak currents observed in the masked devices (Fig. S6B).

Cyclic voltammetry (CV) was employed to assess the electrochemical properties of the immunosensor. CV curves demonstrated distinct changes in current response upon stepwise surface modification (Fig. 1E and F). The bare MWCNT electrode exhibited a peak current of 1691 μA, which increased significantly to 2367 μA following AuNP deposition, highlighting the conductivity enhancement conferred by the AuNPs. Subsequent antibody functionalization (anti-CEA and anti-Ki67) resulted in a decrease in peak current to 1580 μA and 1770 μA, respectively, consistent with the increased resistance to electron transfer upon biomolecule immobilization.

Optimization of the antigen-antibody incubation time revealed saturation of the signal at 35 min for both CEA and Ki67 (Fig. S7). This optimal incubation time was used for all subsequent measurements. To minimize non-specific binding, the antibody-functionalized electrodes were treated with bovine serum albumin (BSA), leading to a further reduction in peak currents. The electrochemical behavior of the AuNP@MWCNT electrode was further investigated by CV at varying scan rates (30–350 mV s^−1^) (Fig. S8A). Both anodic and cathodic peak currents exhibited a linear dependence on the square root of the scan rate (Fig. S8B), indicative of a diffusion-controlled electrochemical process.

Following optimization of the individual sensor components, the complete microfluidic electrochemical immunosensor platform was assembled for *in vitro* biomarker detection. Linear sweep voltammetry (LSV) was used to evaluate the sensor response to varying concentrations of CEA and Ki67 (Fig. 1G and H). In the case of CEA, LSV peak currents (I_p_) exhibited a non-linear decrease with increasing CEA concentration, ranging from 0 to 500 ng/ml. The measured peak currents were normalized according to the following equation (1):

(1) $$\mathrm{Normalized}\;I_{p}=\frac{Measured\;I_{p}}{\;I_{p0}}$$

Where the measured I_p_ was each peak current after treatment of CEA at different concentrations, and the I_p0_ was the peak current of the control sample (*i.e.,* 0 ng/mL CEA). In the case of CEA, LSV peak currents (I_p_) decreased as increasing of CEA concentrations, which indicated the immunosensor could quantitively detect CEA ranging from 0 to 500 ng/mL with a detection limit of 0.97 ng/ml. The decreased peak current indicated that the antigens were bound to immobilized antibodies onto the electrode, suggesting inhibited transfer of electrons of the working electrode [30]. The non-linear decrease in peak current from 125 to 500 ng/mL concentrations of CEA was due to fewer active sites of the antibodies as increasing concentrations of antigens and more formation of antibody–antigen complexes [28]. Similarly, we measured the LSV response after the treatment of Ki67 at different concentrations (0–500 ng/mL). The peak currents non-linearly decreased in proportion to the increase in Ki67 concentration with detection limit of 0.97 ng/mL, as observed in CEA test. The quantitative and effective detection of CEA and Ki67 was attributed to the large surface area and high conductivity of MWCNT@AuNP electrode, which induced better distribution of active sites and enhanced electron transfer [31,32]. Furthermore, to compare the sensing properties of the immunosensor, Table S2 provides previously reported biosensors for detection of CEA or Ki67. Although the LOD of the immunosensor is generally competitive, some sensing platforms exhibited higher sensitivity toward single biomarker detection. However, none of those studies suggested a multiplex sensing platform which is capable of detecting both CEA and Ki67 simultaneously. In contrast to previous works, our immunosensor uniquely incorporated CEA and Ki67 antigen multiplex sensing properties. Together with these results, we observed that the microfluidic platform-based electrochemical immunosensor has the potential to detect cancer markers.

### 3.2 Characterization of mouse-derived tumor spheroid

**Fig. 2:**
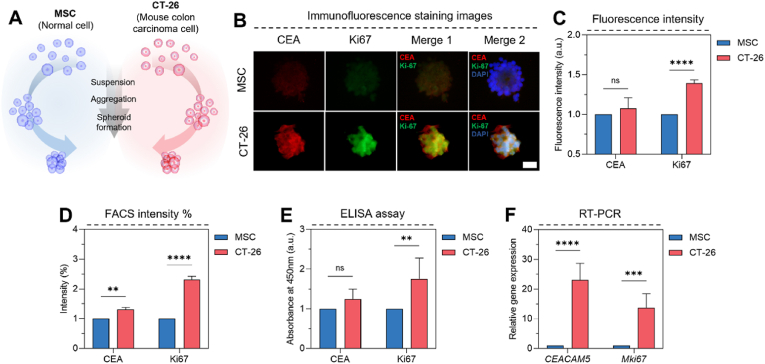
Characterization of mouse-derived tumor spheroid. (A) Schematic diagram of spheroids for CT-26 cells and MSCs. (B) Immunofluorescence staining images of tumor spheroid and normal spheroid (X630). (C) Fluorescence expression area graph in tumor spheroids and normal spheroids (n = 6). (D) Intensity graph in tumor spheroids and normal spheroids for FACS (n = 3). (E) Protein expression by ELISA assay from cultured medium (n = 4). (F) Real-time quantitative PCR graphs of tumor spheroids and normal spheroids (n = 5). Normal cell: MSC (mesenchymal stem cell), Tumor cell: CT-26 (mouse colorectal carcinoma cell). Scale bar: (B) 40 μm. All data represent mean ± SD. *∗p* < 0.05, *∗∗p* < 0.01, *∗∗∗∗p* < 0.0001. The symbol *∗* indicates comparisons with MSC.

To generate 3D tumor spheroids and obtain conditioned culture media for subsequent analysis with our electrochemical immunosensor, CT-26 murine colorectal cancer cells and MSCs were cultured under 3D conditions for 2 days (Fig. S9). This approach allowed us to compare the expression of key cancer-related biomarkers between cancerous and non-cancerous cells in a physiologically relevant 3D microenvironment (Fig. 2A). CEA was first isolated from CRC tissue in 1965 and is a fetal saccharide, which is generally not produced in large quantities after birth [33]. However, there is a shape of increased CEA expression in colon cancer, and preoperative CEA elevation in clinical patients increased the risk of death by 62 % compared to normal CEA levels [34]. In addition, Ki67 is a nuclear proliferation-related protein, and the index of Ki67 influences the recurrence rate and survival rate by the proliferation activity of cancer cells [35]. Therefore, we focused our analysis on CEA, a well-established CRC biomarker, and Ki67, a nuclear protein associated with cell proliferation. A platform was created to detect abnormal proliferation activity of cancer cells through Ki67, a cancer proliferation factor, and to detect specific factors additionally through CEA, a colon cancer biomarker, to clinically diagnose cancer early.

**Fig. 3:**
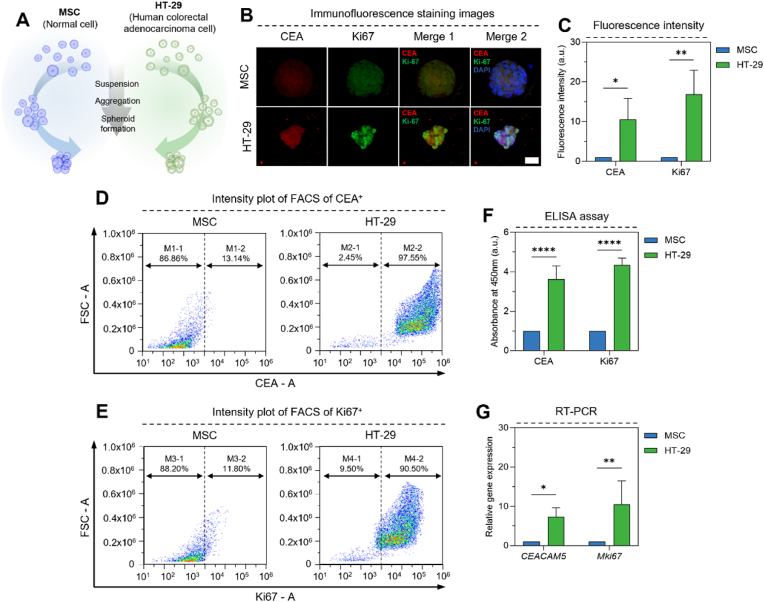
Characterization of human-derived tumor spheroid. (A) Schematic diagram of spheroids for HT-29 cells and MSCs. (B) Immunofluorescence staining images of tumor spheroid and normal spheroid (X630). (C) Fluorescence expression area graph in tumor spheroids and normal spheroids (n = 3). (D) FACS intensity plot of tumor spheroid and normal spheroid for CEA. (E) FACS intensity plot of tumor spheroid and normal spheroid for Ki67. (F) Protein expression by ELISA assay from cultured medium (n = 5). (G) Real-time quantitative PCR graphs of tumor spheroids and normal spheroids (n = 3). Normal cell: MSC (mesenchymal stem cell), Tumor cell: HT-29 (Human colorectal adenocarcinoma cell). Scale bar: (B) 40 μm. All data represent mean ± SD. *∗p* < 0.05, *∗∗p* < 0.01, *∗∗∗∗p* < 0.0001. The symbol *∗* indicates comparisons with MSC.

Immunofluorescence staining of the 3D spheroids revealed distinct differences in CEA and Ki67 expression patterns. While MSC spheroids exhibited diffuse and weak staining for both markers, CT-26 spheroids displayed strong and well-defined staining (Fig. 2B). The quantification of fluorescence intensity confirmed significantly higher expression of CEA (1.08-fold) and Ki67 (1.40-fold) in CT-26 spheroids than in MSC spheroids (Fig. 2C). To further quantify CEA and Ki67 expression, we performed a flow cytometry analysis. The proportion of cells exhibiting high fluorescence intensity (≥1 × 10^3.5^) was significantly higher in CT-26 spheroids compared to MSC spheroids for both CEA (32.88 % vs. 14.68 %) and Ki67 (54.06 % vs. 39.65 %) (Fig. 2D and S10). These results demonstrate a 2.24-fold increase in the expression of both CEA and Ki67 in the CT-26 cells.

An analysis of the spheroid-conditioned culture media by ELISA corroborated the cellular expression data. CEA levels were 1.24-fold higher in the CT-26 conditioned media compared to the MSC media, while Ki67 levels were 1.75-fold higher (Fig. 2E). Furthermore, RT-PCR analysis of the cells themselves revealed a substantial upregulation of *CEACAM5* (21.15-fold) and *Mki67* (12.40-fold) mRNA expression in CT-26 cells compared to MSCs (Fig. 2F). Taken together, these findings demonstrate that CT-26 murine colorectal cancer cells exhibit significantly higher expression of CEA and Ki67, both at the cellular and protein level, compared to non-cancerous MSCs. This differential expression was consistently observed across multiple analytical techniques, including immunofluorescence, flow cytometry, ELISA, and RT-PCR, and was also reflected in the conditioned culture media. These results highlight the potential of CEA and Ki67 as diagnostic biomarkers for CRC and validate the suitability of the 3D spheroid model for studying biomarker expression.

### 3.3 Characterization of human-derived tumor spheroid

To extend our findings to a human model, we generated 3D spheroids using the HT-29 human colorectal cancer cell line (Fig. 3A). As with the murine model, we compared biomarker expression in HT-29 spheroids to that of MSC spheroids. Immunofluorescence staining revealed a striking difference in CEA and Ki67 expression. HT-29 spheroids displayed robust and well-defined staining for both markers, while MSC spheroids exhibited weak and diffuse staining (Fig. 3B). Quantitative analysis of fluorescence intensity demonstrated a remarkable increase in CEA (10.56-fold) and Ki67 (16.88-fold) expression in HT-29 spheroids compared to MSC spheroids (Fig. 3C). Flow cytometry analysis further confirmed the elevated expression of CEA and Ki67 in HT-29 cells. The proportion of cells with high fluorescence intensity (≥1 × 10^4^) was substantially greater in HT-29 spheroids compared to MSC spheroids for both CEA (90.50 % vs. 13.14 %) and Ki67 (97.55 % vs. 11.80 %) (Fig. 3D–E and S11). Analysis of the mean fluorescence intensity (MFI) revealed a 17.04-fold increase in CEA expression and a 4.58-fold increase in Ki67 expression in HT-29 cells compared to MSCs. ELISA analysis of the spheroid-conditioned culture media supported the cellular expression data. CEA levels were 3.62-fold higher in the HT-29 conditioned media compared to the MSC media, while Ki67 levels were 4.35-fold higher (Fig. 3F). RT-PCR analysis of the cells themselves showed a significant upregulation of *CEACAM5* (7.33-fold) and *Mki67* (10.52-fold) mRNA expression in HT-29 cells compared to MSCs (Fig. 3G).

These results collectively demonstrate that the human-derived HT-29 colorectal cancer cells exhibit significantly higher expression of CEA and Ki67 compared to non-cancerous MSCs. This differential expression was consistently observed across multiple analytical platforms, including immunofluorescence, flow cytometry, ELISA, and RT-PCR. Biomarker expression between HT-29 cells and MSC was larger than that observed in the murine CT-26 model. When compared with CEA and Ki67 in this normal range, it can be seen that the biomarker expression of human CRC was more clearly shown. These findings further validate the suitability of our 3D spheroid model for studying biomarker expression and lay the groundwork for evaluating the performance of our microfluidic electrochemical immunosensor in detecting these tumor-related factors.

### 3.4 Characterization of transfected human-derived tumor spheroid

**Fig. 4:**
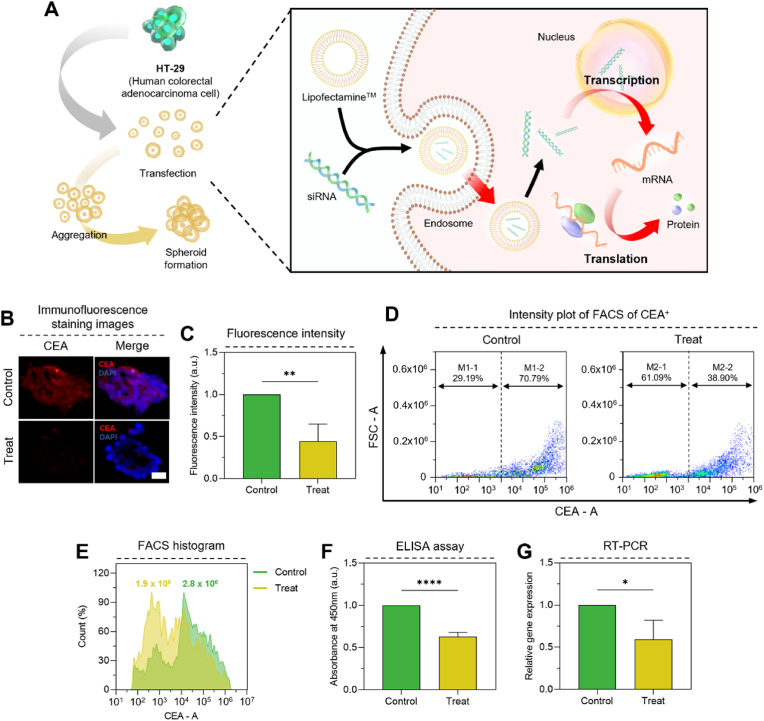
Characterization of transfected human-derived tumor spheroid. (A) Schematic diagram of spheroids for transfected HT-29 cells. (B) Immunofluorescence staining images of transfected spheroids (X630). (C) Fluorescence expression area graph in spheroids (n = 6). (D) Intensity plots of transfected spheroid for FACS. (E) Histogram plot HT-29 cells and transfected HT-29 cells. (F) Protein expression by ELISA assay from cultured medium (n = 5). (G) Real-time quantitative PCR graphs of spheroids (n = 3). Control: HT-29 spheroids (non-transfected), Treat: transfected HT-29 spheroids. Scale bar: (B) 40 μm. All data represent mean ± SD. *∗p* < 0.05, *∗∗p* < 0.01, *∗∗∗∗p* < 0.0001. The symbol *∗* indicates comparisons with Control.

Having established that CEA and Ki67 are highly expressed in colorectal cancer spheroids and their conditioned media, we next sought to confirm that the observed CEA expression was specifically derived from the tumor cells themselves. To this end, we performed siRNA-mediated knockdown of *CEACAM5* (the gene encoding CEA) in HT-29 cells and subsequently generated 3D spheroids from these transfected cells (Fig. 4A).

Immunofluorescence staining of the transfected spheroids revealed a marked reduction in CEA expression compared to non-transfected control spheroids (Fig. 4B). Quantitative analysis of fluorescence intensity confirmed a significant decrease (0.44-fold) in CEA expression in the transfected spheroids (Fig. 4C). Flow cytometry analysis further validated the efficacy of *CEACAM5* knockdown. The proportion of cells exhibiting high fluorescence intensity (≥1 × 10^4^) was significantly reduced in the transfected spheroids (38.90 %) compared to the control spheroids (70.79 %) (Fig. 4D). Furthermore, the MFI of CEA was reduced by a factor of 0.68 in the transfected spheroids (Fig. 4E). Analysis of the conditioned media from the transfected spheroids by ELISA demonstrated a 0.63-fold decrease in CEA levels compared to the control media (Fig. 4F). RT-PCR analysis of the transfected cells confirmed a 0.60-fold reduction in *CEACAM5* mRNA expression (Fig. 4G).

These results consistently demonstrate successful knockdown of *CEACAM5* and a corresponding reduction in CEA expression at both the cellular and protein levels. This confirms that the CEA detected in our previous experiments was indeed derived from the tumor cells themselves, validating the specificity of CEA as a biomarker for CRC in our 3D spheroid model.

### 3.5 Selectivity of the electrochemical immunosensor

To evaluate the target-specific sensing behavior of the microfluidic electrochemical immunosensor, various biomarkers including the target molecule and interfering substances such as CA19-9, BSA, CEA and Ki67 were tested. Both anti-CEA and anti-Ki67 antibody functionalized immunosensor were incubated with 1 μg/mL of each biomarker for 35 min at 4 °C. A total of three separate immunosensors were prepared for the test. After incubating, the LSV method (ranging from −0.5 V to 0.5 V) was performed to examine the electrochemical properties of the immunosensor. The peak currents were normalized to those of the target markers (CEA for anti-CEA and Ki67 for anti-Ki67 antibody). In the case of anti-CEA, the normalized peak currents were 1.68 ± 0.21, 1.61 ± 0.03 and 1.59 ± 0.13 for BSA, CA19-9 and Ki67, respectively (Fig. S12A). In addition, for anti-Ki67, the normalized peak currents were 1.49 ± 0.12, 1.44 ± 0.15 and 1.50 ± 0.26 for BSA, CA19-9 and CEA, respectively (Fig. S12B). Based on this result, it was confirmed that both anti-CEA and anti-Ki67 functionalized immunosensors showed clear selectivity toward target biomarker toward other interfering molecules including non-specific protein (*i.e.* BSA), and other cancer biomarker (*i.e.* CA19-9) with a noticeable difference of up to 67.87 % in the peak current value. This result demonstrates that the immunosensors clearly distinguished target biomarkers without cross-reactivity.

### 3.6 Detection of CEA and Ki67 in spheroid medium

**Fig. 5:**
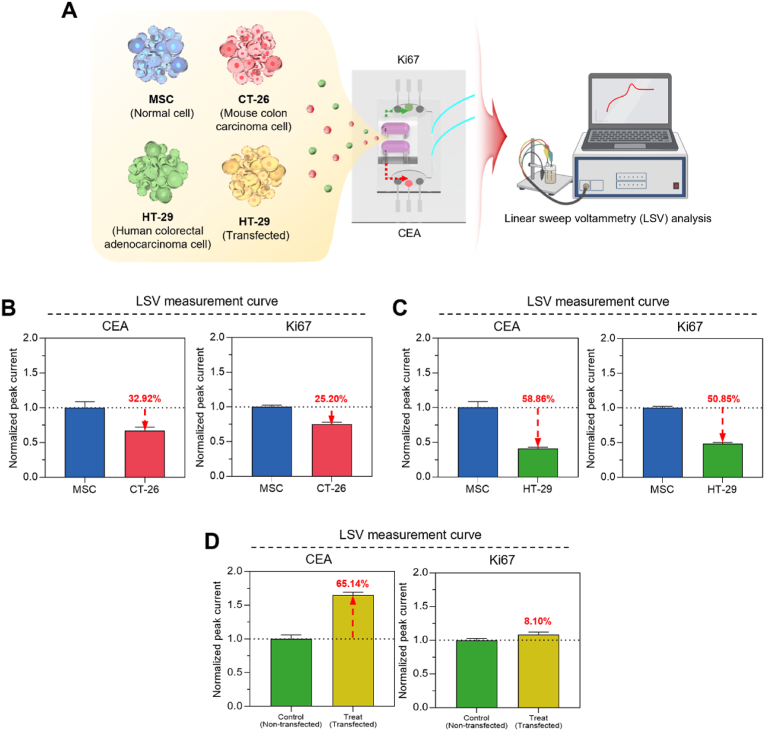
Detection of CEA and Ki67 in spheroid medium. (A) Schematic illustrations of antigen detection from CT-26, MSC and HT-29 culture medium using the immunosensor. LSV measurements comparison of anti-CEA antibodies and anti-Ki67 antibodies functionalized immunosensor for (B) CT-26 vs MSC, (C) HT-29 vs MSC, and (D) Treat (Transfected) vs Control (Non-transfected) after incubating in various cell culture medium with a voltage sweep from −0.5 V to 0.5 V at a 50 mV/s scan rate (n = 3).

To evaluate the performance of our microfluidic electrochemical immunosensor in a biologically relevant context, we analyzed conditioned media from 3D spheroids generated from CT-26 murine colorectal cancer cells, HT-29 human colorectal cancer cells, and MSCs (Fig. 5A). LSV measurements revealed a significant decrease in peak current upon incubation with CT-26 spheroid-conditioned media compared to MSC media, with reductions of 32.92 % ± 4.94 % and 25.20 ± 3.18 % observed for the CEA and Ki67 sensors, respectively (Fig. 5B). This decrease in peak current is consistent with the higher levels of CEA and Ki67 in the CT-26 media, which bind to the antibodies on the sensor surface and hinder electron transfer.

Similarly, analysis of HT-29 spheroid-conditioned media demonstrated a substantial reduction in peak current compared to MSC media, with decreases of 58.86 ± 1.77 % and 51.46 ± 1.91 % observed for the CEA and Ki67 sensors, respectively (Fig. 5C). These results confirm that both CEA and Ki67 are more abundantly expressed in HT-29 spheroids compared to CT-26 spheroids and further demonstrate the ability of our microfluidic electrochemical immunosensor to detect these biomarkers in different types of spheroid-conditioned media effectively.

To further assess the versatility of our immunosensor, we investigated its ability to detect changes in protein expression following siRNA-mediated knockdown. HT-29 spheroids were treated with siRNA targeting *CEACAM5*, and the conditioned media from these transfected spheroids were analyzed using our immunosensor. LSV measurements revealed a 65.14 ± 4.38 % increase in peak current for the CEA sensor upon incubation with siRNA-treated media compared to control media (Fig. 5D). This increase in peak current is consistent with the reduced levels of CEA in the siRNA-treated media, confirming the successful knockdown of *CEACAM5* and demonstrating the sensitivity of our sensor to changes in CEA expression. Importantly, the peak current for the Ki67 sensor did not exhibit a significant change (8.10 ± 3.93 % increase) upon incubation with siRNA-treated media. This result indicates that the siRNA treatment specifically targeted CEA expression without affecting Ki67 expression, further highlighting the specificity of our immunosensor. Moreover, the consistent detection of Ki67 in both control and siRNA-treated media demonstrates the robustness of our sensor and its ability to function effectively in complex biological samples.

Taken together, these findings demonstrate the potential of our microfluidic electrochemical immunosensor for detecting and quantifying CEA and Ki67 in 3D spheroid-conditioned media. The ability of our sensor to detect changes in biomarker expression following siRNA-mediated knockdown further underscores its utility for monitoring therapeutic responses and studying the dynamics of biomarker expression in cancer models.

### 3.7 Repeatability and stability of the immunosensor

The repeatability for both anti-CEA and anti-Ki67 functionalized immunosensors was studied using the LSV method. The immunosensors were incubated with 1 μg/mL of the target biomarker for 35 min (CEA for anti-CEA and Ki67 for anti-Ki67), and then, three independent measurements were performed for three separate immunosensors. The LSV curves were normalized to the peak current value of each immunosensor, as shown in Figs. S13A and S13B. For both anti-CEA and anti-Ki67, the immunosensors showed +4.11 %/– 6.00 % difference (±5.08 % standard deviation) and +3.15 %/– 6.04 % difference (±4.67 % standard deviation) relative to the median value, respectively. This result indicates that both anti-CEA and anti-Ki67 immunosensors have consistent repeatability. Further, the long-term stability was investigated for both anti-CEA and anti-Ki67 functionalized immunosensors after reacting with 1 μg/mL of the target biomarker. The immunosensors were stored at 4 °C when not in use for measurement and tested after various storage times. In total, seven data points were collected over a time span ranging from 0 to 232.1 h and 0–238.5 h for anti-CEA and anti-Ki67, respectively. LSV measurements were performed to analyze the stability, and peak current data were normalized to those at 0 h storage time. In the case of anti-CEA, the normalized peak current values were measured as 0.984 (84.4 h), 0.996 (116.4 h), 1.014 (169.9 h), 0.988 (182.9 h), 0.926 (210.1 h) and 0.994 (232.1 h) (Fig. S13C). Moreover, the normalized peak current values were 0.973 (90.8 h), 0.960 (122.8 h), 0.941 (169.3 h), 0.918 (189.3 h), 0.952 (216.5 h) and 0.863 (238.5 h) for anti-Ki67 (Fig. S13D). The peak current values displayed a slight decrease overall compared to those at 0 h storage time, with however, the variation was negligible with low standard deviation which was calculated as ± 2.799 % and ±4.370 % for anti-CEA and anti-Ki67, respectively. This result indicates that the immunosensors showed reliable long-term stability.

## 4 Conclusion

In this study, we developed a novel microfluidic-based electrochemical immunosensor platform for the sensitive and specific detection of CEA and Ki67, two important biomarkers in colorectal cancer. The platform was fabricated using a cost-effective and scalable approach, combining 3D printing for the microfluidic layer and PCB printing for the AuNP@MWCNT electrodes. The resulting immunosensor exhibited a low detection limit of 0.97 ng/ml for both CEA and Ki67, enabling quantitative analysis of these biomarkers in complex biological samples. Prior to evaluating the sensor's performance, we rigorously characterized the expression of CEA and Ki67 in 3D spheroids generated from murine (CT-26) and human (HT-29) colorectal cancer cell lines, as well as non-cancerous mesenchymal stem cells (MSCs). Using a combination of techniques, including flow cytometry, immunofluorescence staining, ELISA, and RT-PCR, we confirmed significantly elevated expression of both biomarkers in the cancer cell lines compared to the MSCs. Importantly, the electrochemical measurements obtained with our immunosensor were consistent with these expression patterns, validating its reliability and accuracy. Furthermore, we demonstrated the ability of our immunosensor to monitor changes in CEA expression following siRNA-mediated knockdown of *CEACAM5* in HT-29 spheroids. This finding highlights the potential of our platform for studying dynamic changes in biomarker expression and evaluating therapeutic responses in preclinical models. Based on these results, our microfluidic-based electrochemical immunosensor enables multiplexed analysis using a single platform. The simple manufacturing process and low sample volume requirement make the sensor suitable for point-of-care applications and portable diagnostic systems. Moreover, incorporating multiplexed screening into electrochemical readout ensures rapid and sensitive measurements. This advantage offers the potential of highly specific and accurate analysis for not only biomarker expressions in cell line or spheroid, but also real clinical samples, such as patient-derived serum or tissue fluids. By integrating this technology with 3D spheroid models, which more accurately recapitulate the tumor microenvironment, we provide a powerful tool for cancer research and diagnostics. This platform has the potential to accelerate the discovery of new cancer biomarkers, facilitate the development of targeted therapies, and ultimately improve patient outcomes.

## CRediT authorship contribution statement

**Sujin Kim:** Writing – original draft, Methodology, Investigation. **Seonyeop Kim:** Writing – original draft, Methodology, Investigation. **Chanjin Ko:** Writing – original draft, Methodology, Investigation. **Wonseok Lee:** Writing – review & editing, Writing – original draft, Supervision, Methodology, Investigation, Conceptualization. **Hwan Drew Kim:** Writing – review & editing, Writing – original draft, Supervision, Methodology, Investigation, Conceptualization.

## Funding

This work was supported by the 10.13039/501100014188Ministry of Science and ICT of Korea (grant number NRF-2021R1C1C2004576, NRF-2022R1A2C4001990, RS-2023-00222737) and the Korean Fund for Regenerative Medicine (KFRM) grant, which is funded by the Korean government (the 10.13039/501100014188Ministry of Science and ICT, the 10.13039/100009647Ministry of Health & Welfare) (Code: KFRM 22A0105L1-11).

## Declaration of competing interest

The author is an Editorial Board Member/Editor-in-Chief/Associate Editor/Guest Editor for this journal and was not involved in the editorial review or the decision to publish this article.

The authors declare the following financial interests/personal relationships which may be considered as potential competing interests:Hwan Drew Kim and reports financial support was provided by Ministry of Science and ICT of Korea. Wonseok Lee reports financial support was provided by Ministry of Science and ICT of Korea. Hwan Drew Kim reports financial support was provided by Korean Fund for Regenerative Medicine. If there are other authors, they declare that they have no known competing financial interests or personal relationships that could have appeared to influence the work reported in this paper.

## Data Availability

Data will be made available on request.
