# Supplementary material for: A microfluidic electrochemical immunosensor for detection of CEA and Ki67 in 3D tumor spheroids
Source: Mater Today Bio. 2025 Apr 12;32:101768. doi: 10.1016/j.mtbio.2025.101768 (PMC12022681; doi:10.1016/j.mtbio.2025.101768)
Supplement: Multimedia component 1 [file mmc1.docx]

Supporting Information

**A Microfluidic Electrochemical Immunosensor for Detection of CEA and Ki67 in 3D Tumor Spheroids.**

*Sujin Kim^a,#^, Seonyeop Kim^b,#^, Chanjin Ko^b^, Wonseok Lee^b ,c*^, and Hwan Drew Kim^a, d*^*

^a^ Department of Polymer Science and Engineering, Korea National University of Transportation, 50 Daehak-ro, Chungju, 27469, Republic of Korea

^b^ Department of IT-Energy Convergence (BK21 Four), Chemical Industry Institute, Korea National University of Transportation, 50 Daehak-ro, Chungju, 27469, Republic of Korea

^c^ Department of Electrical Engineering, Korea National University of Transportation

50 Daehak-ro, Chungju, 27469, Republic of Korea

^d^ Department of Biomedical Engineering, Korea National University of Transportation, 50 Daehak-ro, Chungju, 27469, Republic of Korea

^#^ Co-author contributed equally to this work.

^*^Corresponding author: [wslee@ut.ac.kr](mailto:wslee@ut.ac.kr) (W. Lee); [hdkim@ua.ac.kr](mailto:hdkim@ua.ac.kr) (H. D. Kim)


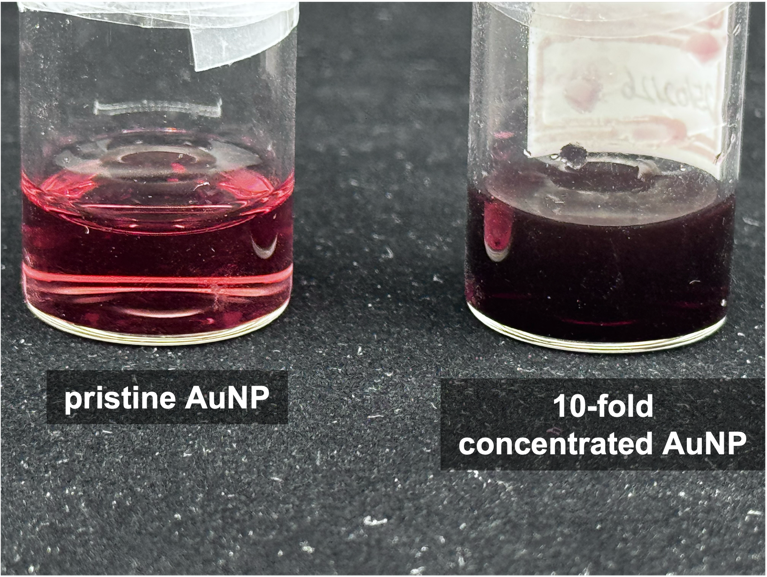


**Fig. S1** Image of fabricated pristine AuNP and 10-fold concentrated AuNP solution.


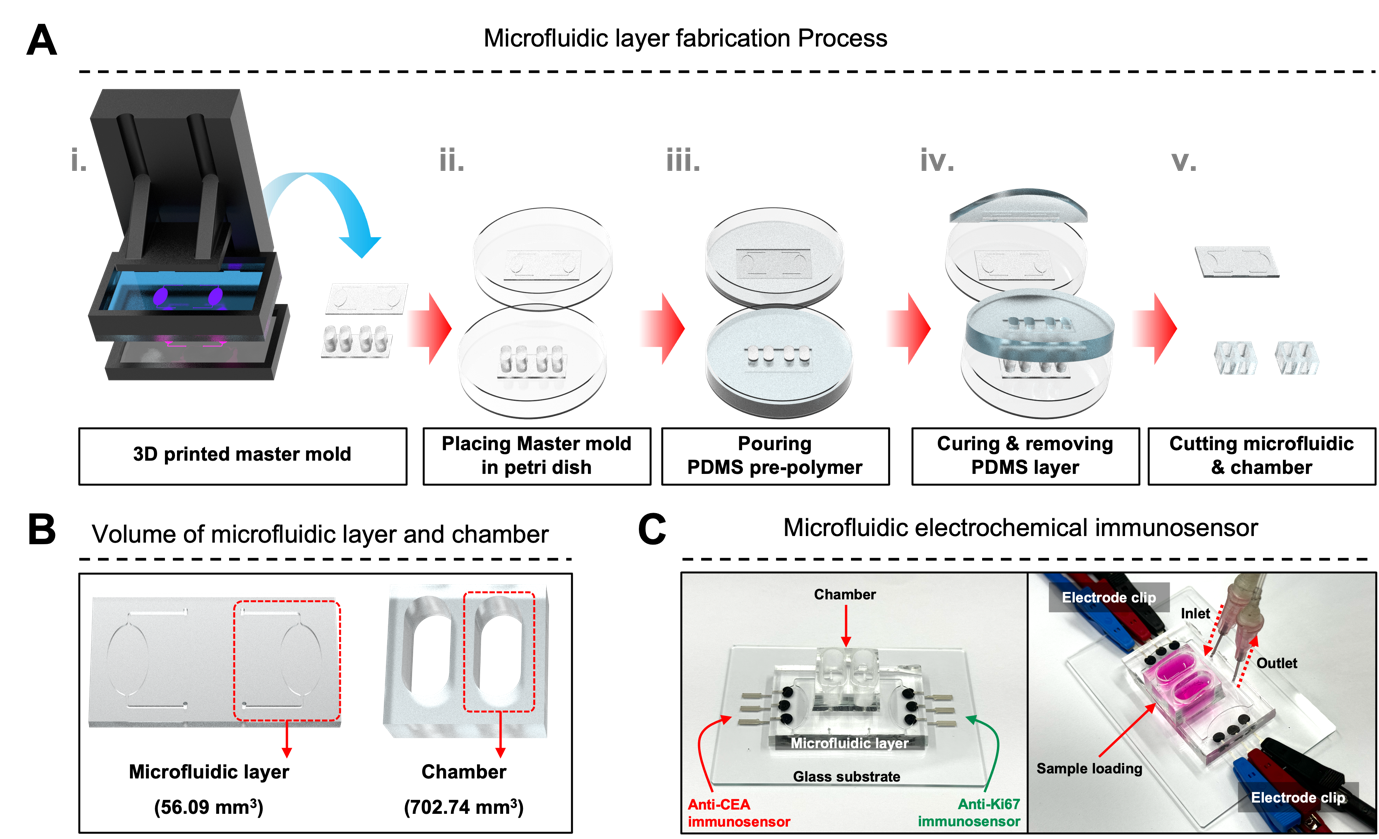


**Fig. S2** Microfluidic layer fabrication process. (A) Fabrication process and (B) schematic illustrations of microfluidic layer and chamber using SLA 3D printing method. (C) Image of fabricated microfluidic electrochemical immunosensor for detection and CEA and Ki67.


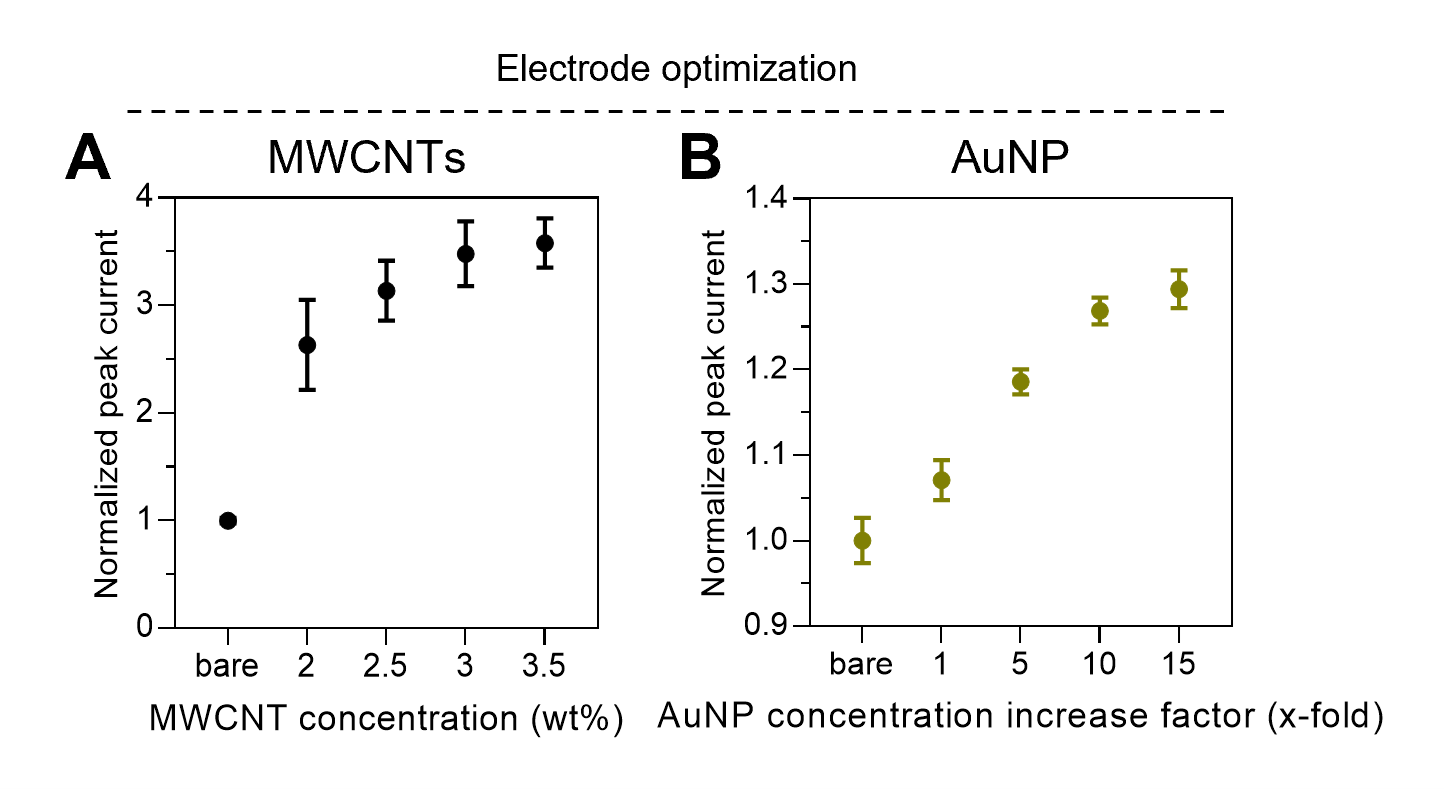


**Fig. S3** Normalized LSV peak currents of various concentrations of (A) MWCNTs and (B) AuNP (n = 3).


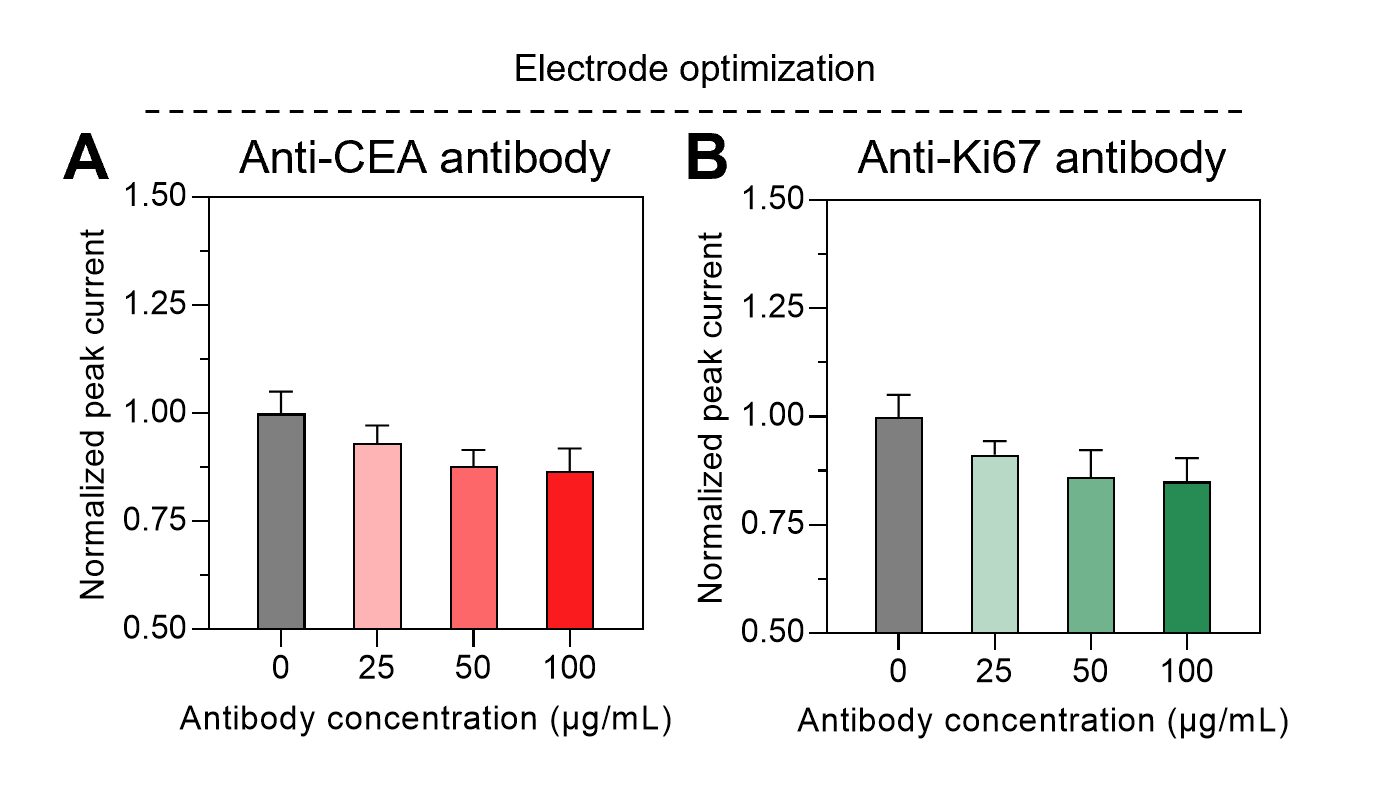


**Fig. S4** Normalized LSV peak currents of various concentrations of (A) anti-CEA and (B) anti-Ki67 antibody (n = 3).


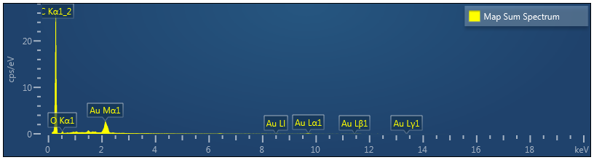


**Fig. S5** EDS spectra of AuNP@MWCNTs electrode.


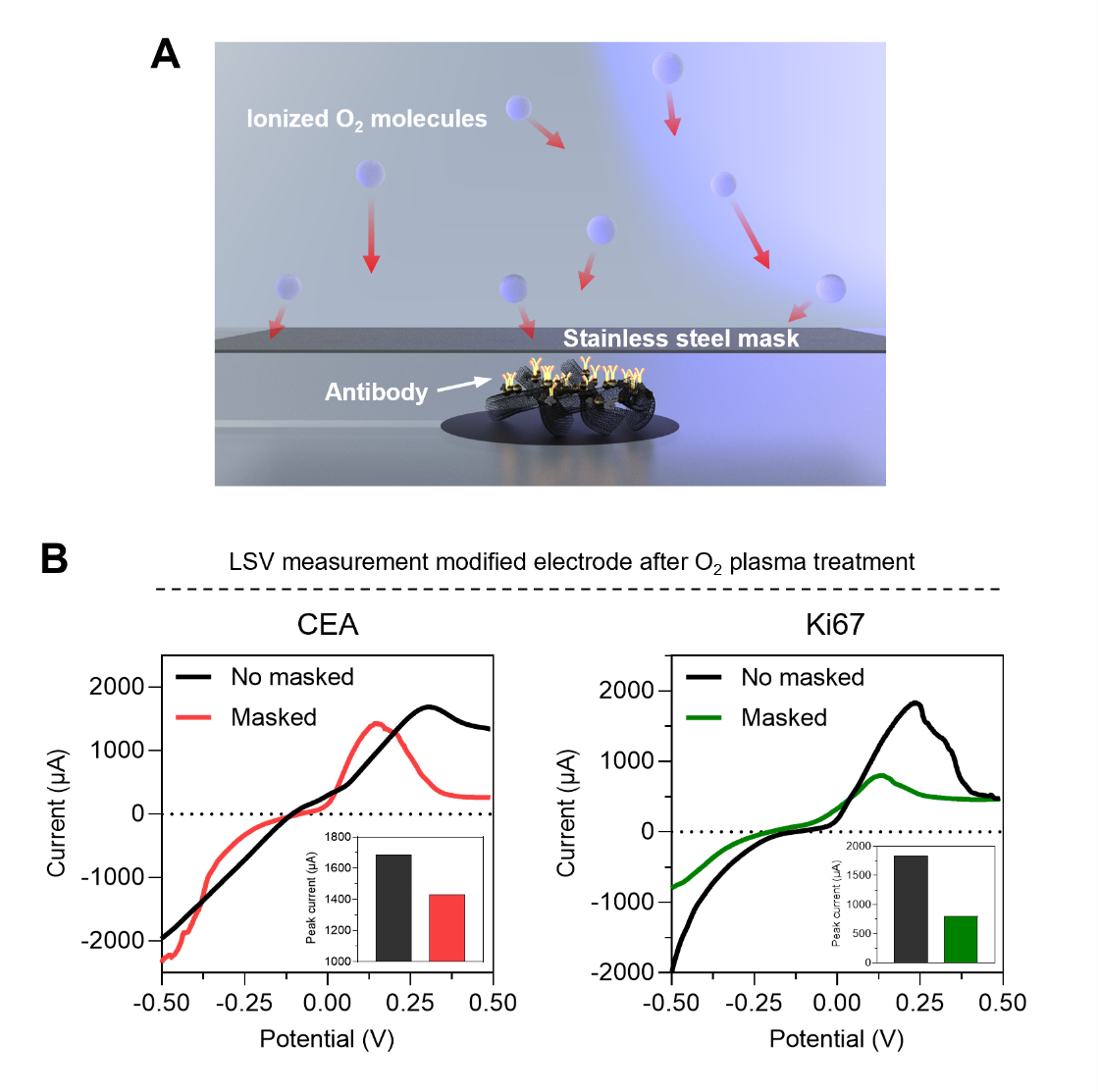


**Fig. S6** Masking process (antibody protection against O_2_ plasma). (A) Schematic illustrations of protection of antibodies onto the electrode against O_2_ plasma treatment using stainless steel (SUS450) mask. (B) – (C) LSV measurement of anti-Ki67 antibody and anti-CEA antibody modified electrode after O_2_ plasma treatment with / without stainless steel mask (voltage sweep from -0.5 V to 0.5 V at 50 mV/s scan rate).


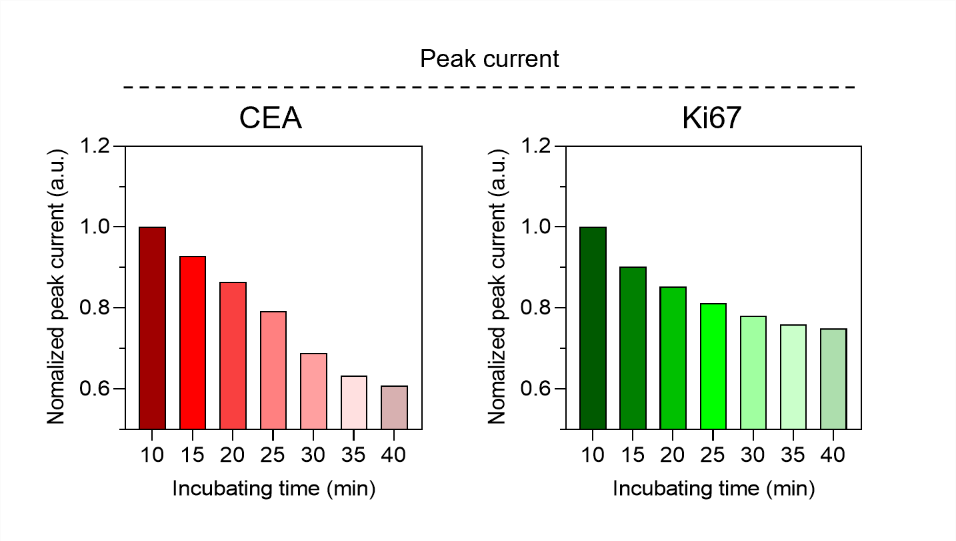


**Fig. S7** Peak current of anti-CEA antibody and anti-Ki67 antibody functionalized MWCNTs@AuNP electrode with various incubating times in CEA (1.953 ng/mL) solution and Ki67 antigen (7.8126 ng/mL), respectively. (voltage range from -0.5 V to 0.5 V at 50 mV/s scan rate and 2.5 mV step).


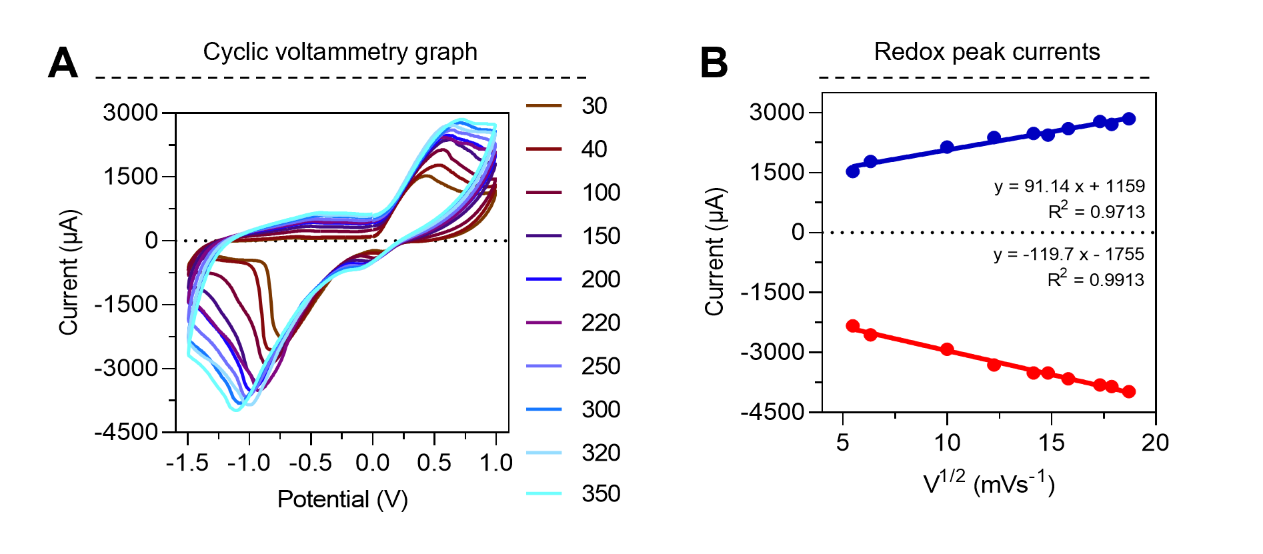


**Fig. S8** Scan rate analysis (A) Cyclic voltammograms of the AuNP@MWCNTs electrode at different scan rates (30, 40, 100, 150, 200, 220, 250, 300 and 350 mV/s in 5 mM Fe(CN)_6_^3-/4-^ + 0.1 M KCl + 0.01 M PBS solution at room temperature condition. (B) Dependence of the redox peak currents on the square root of scan rates.


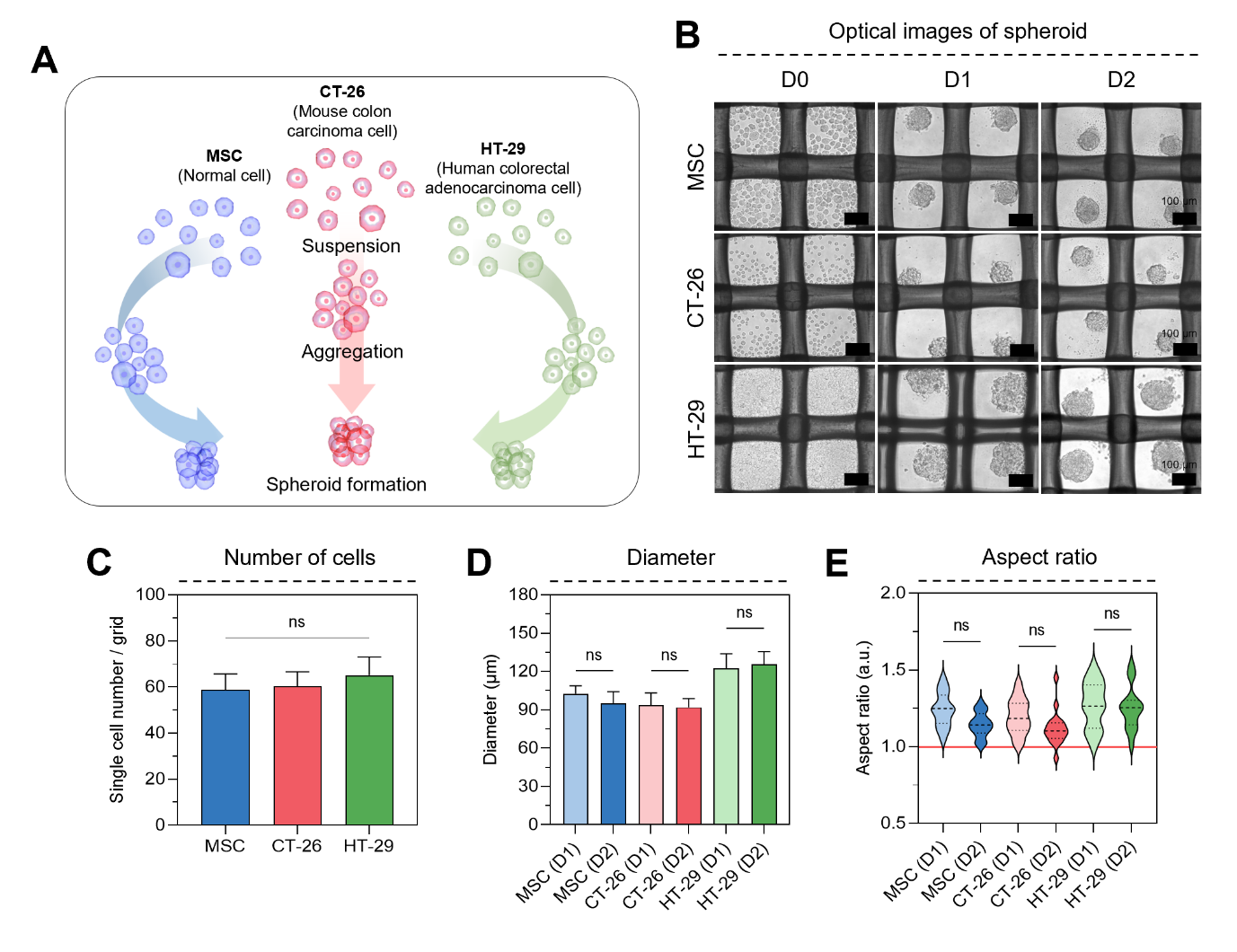


**Fig. S9** Fabrication of tumor spheroids. (A) Schematic diagram of spheroids for MSCs, CT-26 cells and HT-29 cells. (B) Optical images of normal spheroids and tumor spheroids (X200). (C) Graph of the number of cells in the grid (n = 3). (D) Diameter graph of tumor spheroids and normal spheroids on day 1 and day 2 (n = 3). (E) Aspect ratio graph of tumor spheroids and normal spheroids on day 1 and day 2 (n = 3). Normal cell: MSC (mesenchymal stem cell), Tumor cell: CT-26 (colorectal carcinoma cell), HT-29 (Human colorectal adenocarcinoma cell).


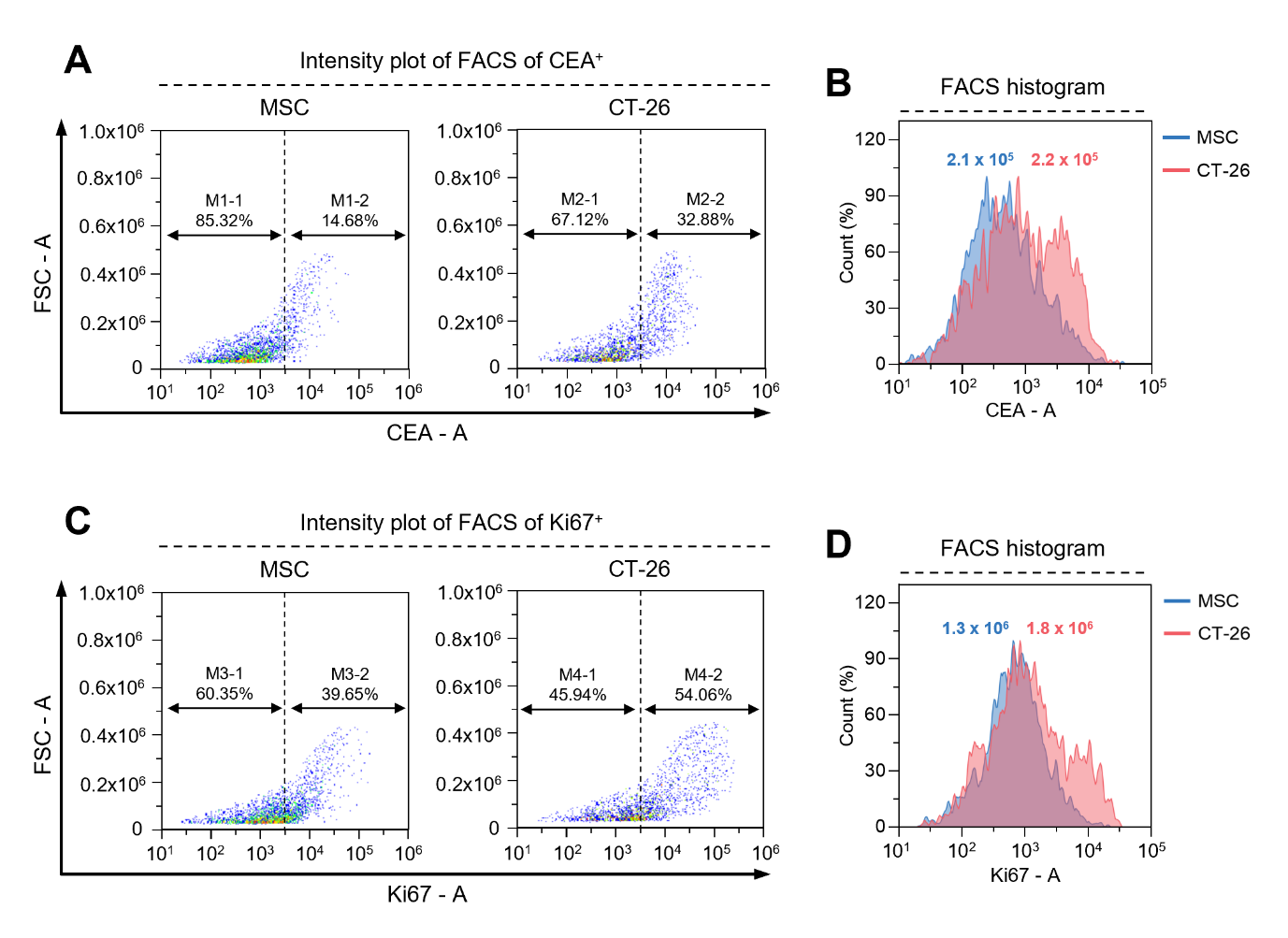


**Fig. S10** FACS intensity plot of tumor spheroid and normal spheroid for (A), (C) Intensity plot of MSC and CT-26 spheroid for FACS. (B), (D) Histogram plot of MSC and CT-26 spheroid.


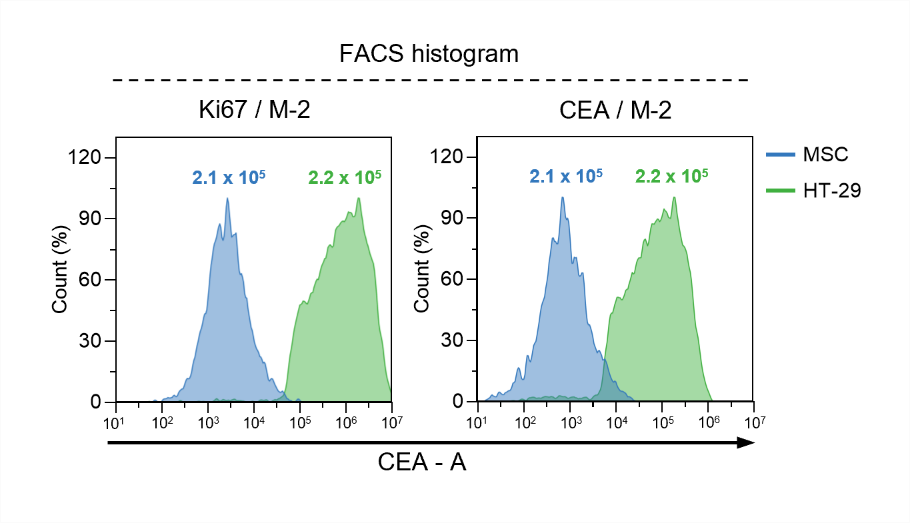


**Fig. S11** Histogram plot MSCs and HT-29 cells.


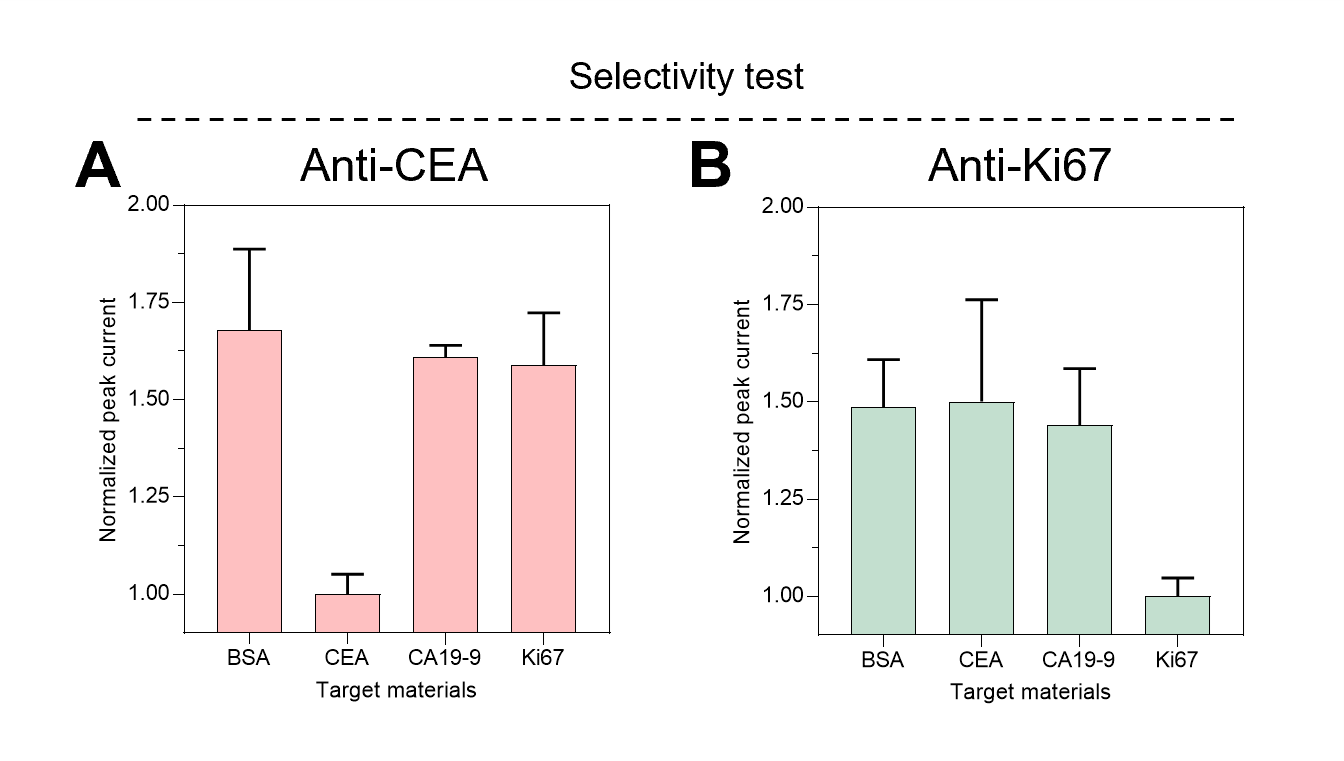


**Fig. S12** Selectivity test of (A) anti-CEA and (B) anti- Ki67 antibody functionalized electrochemical immunosensor toward various biomarkers (BSA, CA19-9, CEA and Ki67) with normalized peak currents from LSV measurements (n = 3).


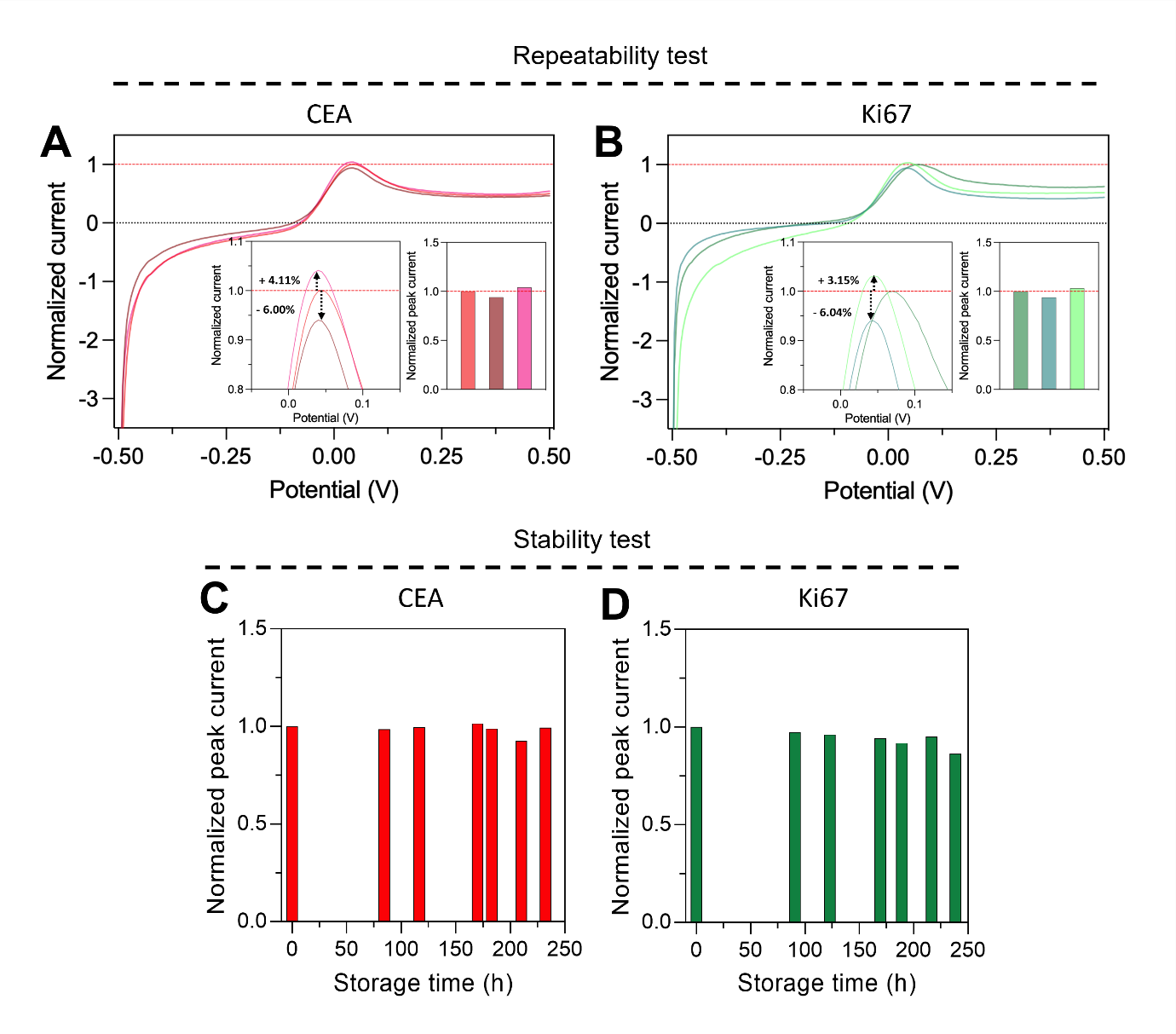


**Fig. S13** Performance reliability of sensors during repeated use. (A) Repeatability test of anti-CEA and anti-Ki67 functionalized immunosensor after reacting with 1 µg/mL of target biomarker. Three independent measurements were conducted using three separate immunosensor. (B) Stability test of anti-CEA and anti-Ki67 functionalized immunosensor after reacting with 1 µg/mL of target biomarker with various storage times.

**Table. S1** The primer sequences.

| **Target** | **Sequence (5’-3’)** |
| --- | --- |
| Mki67  (Mouse) | F: GGACCCTTGCTGTCTTATATTG |
|  | R: AATGATGGCTTTCCCACTAGC |
| CEACAM5  (Mouse) | F: TGTTTGACTCTCACTGGTGG |
|  | R: TGGACAGGGCTTCCTGATAC |
| Mki67  (Human) | F: AGCTACCTGTGGAAAGTAAGAG |
|  | R: AGTAGTGTTGCCTTCTGACC |
| CEACAM5  (Human) | F: GTGGCCCTTCTACAATCAGG |
|  | R: CTCCCGAAAGGTAAGACGAG |
| CEACAM5  (siRNA) | F: AGCTACCTGTGGAAAGTAAGAG |
|  | R: AGTAGTGTTGCCTTCTGACC |

**Table. S2** Analytical performance comparison of CEA and Ki67 detection.\

| **Electrode/Material** | **Methods** | **LOD**  **(ng/mL)** | | **Biomarker** | | **Ref.** |
| --- | --- | --- | --- | --- | --- | --- |
|  |  | **CEA** | **Ki67** | **CEA** | **Ki67** |  |
| AuNPs | Colorimetric | 3 | - | O |  | [1] |
| HRP^@^ ConA/CEA/MCH-Apt/gold electrodes | DPV | 3.4 | - | O |  | [2] |
| Magnetic Core-shell/Fe3O4@Ag/Nafion/MCPE | EIS/CV | 0.5 | - | O |  | [3] |
| AuNP/glass fiber | Colorimetric | 0.45 | - | O |  | [4] |
| GR/MBs-Ab1/CEA/Ab2-AuNPs-HRP | CV | 5 |  | O |  | [5] |
| Lum@SA-AuNP | ECL  (Electrochemiluminescence) | 2.51 |  | O |  | [6] |
| PEI-AuNPs/2D WS_2_/GO | SWV | - | 0.2 |  | O | [7] |
| AuNPs/MWCNT | LSV | 0.97 | 0.97 | O | O | This study |

**Reference**

[1] Luo, C.*, et al.*, *RSC Advances* (2015) **5** (15), 10994

[2] Wang, Q.-L.*, et al.*, *Sensors and Actuators B: Chemical* (2018) **260**, 48

[3] Tang, D.*, et al.*, *The Journal of Physical Chemistry B* (2006) **110** (24), 11640

[4] Tsogka, I.*, et al.*, *Analytical Methods* (2024) **16** (18), 2921

[5] Jin, B.*, et al.*, *Biosensors and Bioelectronics* (2014) **55**, 464

[6] Mohammadniaei, M.*, et al.*, *Talanta* (2024) **266**, 125087

[7] Kuntamung, K.*, et al.*, *Bioelectrochemistry* (2024) **160**, 108780
